# One-Pot Multi-Enzymatic Synthesis of the Four Stereoisomers of 4-Methylheptan-3-ol

**DOI:** 10.3390/molecules22101591

**Published:** 2017-09-22

**Authors:** Elisabetta Brenna, Michele Crotti, Francesco G. Gatti, Daniela Monti, Fabio Parmeggiani, Andrea Pugliese

**Affiliations:** 1 Politecnico di Milano, Dipartimento di Chimica, Materiali, Ingegneria Chimica, Via Mancinelli 7, I-20131 Milano, Italy; michele.crotti@polimi.it (M.C.); francesco.gatti@polimi.it (F.G.G.); fabio.parmeggiani@polimi.it (F.P.); andrea.pugliese@polimi.it (A.P.); 2 Istituto di Chimica del Riconoscimento Molecolare—CNR, Via M. Bianco 9, I-20131 Milano, Italy; daniela.monti@icrm.cnr.it

**Keywords:** pheromone, stereoselectivity, biocatalysis, ene-reductase, alcohol dehydrogenase

## Abstract

The use of pheromones in the integrated pest management of insects is currently considered a sustainable and environmentally benign alternative to hazardous insecticides. 4-Methylheptan-3-ol is an interesting example of an insect pheromone, because its stereoisomers are active towards different species. All four possible stereoisomers of this compound were prepared from 4-methylhept-4-en-3-one by a one-pot procedure in which the two stereogenic centres were created during two sequential reductions catalysed by an ene-reductase (ER) and an alcohol dehydrogenase (ADH), respectively.

## 1. Introduction

The control of native and invasive insects represents an increasingly urgent need as a consequence of the changing climate, the increasing of season temperatures, and the altered rainfall patterns [1]. The thrust to green and sustainable insect control is produced not only by the concern about the health of rural workers, but also by the necessity to improve food security for a growing population while reducing the loss of environmental resources and biodiversity. The idea of replacing hazardous insecticides with environmentally benign and species-specific semiochemicals (i.e., chemical compounds produced by one organism in order to cause a behavioural change in an individual of the same or a different species) is a current research challenge [2]. Semiochemicals which are used in intraspecies communication are called pheromones; when interspecies communication is produced, they are known as allelochemicals. Most of them are nontoxic to vertebrates, and they are released in the environment in low amounts.

The most important applications of this strategy are related to the use of pheromones in the integrated pest management of insects according to the following techniques: (i) monitoring a population of insects to determine if they are present in a certain area or to determine if enough insects are present to require a treatment; (ii) removal of large numbers of insects from the breeding and feeding population by mass trapping; (iii) disruption of mating in populations of insects by dispersing synthetic pheromone into crops to attract males away from females that are waiting to mate [3].

Many pheromones are enantiomerically enriched chiral compounds, since living organisms employ chirality to expand and diversify their communication systems. The absolute configuration of many naturally occurring pheromones has been established, and the relationship between stereochemistry and bioactivity has been extensively studied [4]. The use of chiral pheromones in effective pest management campaigns requires the optimization of scalable and cost-effective synthetic procedures to prepare the necessary stereoisomers in high enantiomeric purity and chemical yield, using readily available starting materials.

4-Methylheptan-3-ol (**1**) represents an interesting example of an insect pheromone whose stereoisomers are active for different species. (3*S*,4*S*)-**1** [5] is one of the components of the aggregation pheromone of the smaller European elm bark beetle (*Scolytus multistriatus* Marsham): it is produced when unmated female beetles bore into American elm. Male large European elm bark beetles (*S. scolytus*) were found to produce traces of (3*S*,4*S*)-**1** and (3*R*,4*S*)-**1**, while females produced only tiny quantities of the (3*R*,4*S*)-**1** [6]. (3*S*,4*S*)-**1** was also identified as the main component of the aggregation pheromone of the almond bark beetle, *S. amygdali* Geurin-Meneville, together with (3*S*,4*S*)-4-methylhexan-3-ol, which acts as a synergist [7]. In field tests, (3*S*,4*S*)-**1** attracted beetles in combination with the synergist (3*S*,4*S*)-4-methylhexan-3-ol, whereas (3*R*,4*S*)-**1** and (3*R*,4*R*)-**1** were inhibitory [8]. Furthermore, (3*R*,4*S*)-**1** was discovered to be the trail pheromone of a southeast Asian ant, *Leptogenis diminuta* of the subfamily Ponerinae [9].

**Scheme 1:**
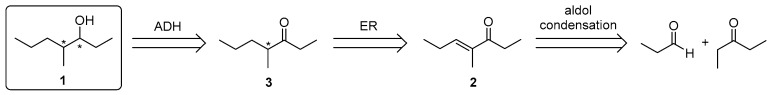
Retrosynthetic approach to the stereoisomers of compound **1**. ADH: alcohol dehydrogenase; ER: ene-reductase.

Herein we report on a very effective enzymatic approach for the enantioselective synthesis of all four stereoisomers of compound **1**, starting from unsaturated ketone **2** (Scheme 1), easily obtained by mixed aldol condensation. The synthetic approach is based on a two-step one-pot multi-enzymatic procedure for the sequential creation of the stereogenic centres under the stereochemical control of two enzymes: an ene-reductase (ER) for the reduction of the C=C double bond and an alcohol dehydrogenase (ADH) for the creation of the alcohol moiety.

## 2. Results

### 2.1. Known Synthetic Routes

The synthetic routes of pheromone **1** reported in the literature are directed mainly to the (3*S*,4*S*)-stereoisomer, taking advantage of a wide range of techniques: (i) enzymatic resolution of *threo*-2-amino-3-methylhexenoic acid, and subsequent manipulation [10]; (ii) asymmetric induction with boronic esters [11]; (iii) chirality transfer by ester enolate Claisen rearrangement [12]; (iv) asymmetric [2,3]-Wittig rearrangement [13]; (v) stereoselective synthesis catalysed by either baker’s yeast [14,15], or chiral palladium-phosphine catalysts [6]; (vi) Sharpless epoxidation followed by regioselective epoxide opening with trimethylaluminum [16].

Five methods have been described for the preparation of all four stereoisomers of compound **1**: (i) HPLC separations of the corresponding Mosher esters on analytical scale (stereoisomeric purity = 94–98%) [17]; (ii) sequential creation of the two stereocentres with diastereoselectivity >99% by using chiral boronic esters [18]; (iii) separation of (3*RS*,4*RS*)-**1** and (3*RS*,4*SR*)-**1** by fractional crystallization of the corresponding 3,5-dinitrobenzoate esters, followed by hydrolysis and subsequent lipase-mediated transesterification of each racemic diastereoisomer with *Candida antarctica* lipase B, using *S*-ethyl octanethioate as an acyl donor (stereoisomeric purity = 61–93%) [19]; (iv) reduction of 3-propyl-4-thianone to yield easily separable *cis*- and *trans*-3-propyl-4-thianols, submitted to resolution by chromatographic separation of the corresponding esters with (*S*)-chlorofluoroacetic acid, followed by hydrolysis and desulfurization (stereoisomeric purity = 91–94%) [20]; (v) preparation of (*R*)- and (*S*)-4-methylheptan-3-ones using SAMP and RAMP reagents, reduction to the corresponding alcohols, and enantioselective transesterification with vinyl acetate catalysed by lipase AK (stereoisomeric purity = 89–95%) [8].

We devised a new synthetic approach to the stereoisomers of **1** starting from unsaturated ketone **2** (Scheme 1), easily obtained in 56% isolation yield by aldol condensation of propanal and 3-pentanone in the presence of potassium hydroxide, followed by dehydration with anhydrous oxalic acid in refluxing toluene. The conversion of **2** into the four stereoisomers of **1** requires the optimization of the enantioselective reduction of both the alkene and the carbonyl group, with either (*R*)- or (*S*)-enantioselectivity.

Biocatalysis offers appealing solutions for this kind of reaction. The enantioselective reduction of the alkene bond can be catalysed by an ER which promotes the stereospecific *anti* hydrogen addition to the C=C double bond, with the delivery of a hydride ion from the reduced flavin mononucleotide prosthetic group, which is restored at the expense of the oxidation of NADPH to NADP^+^ [21,22]. To avoid the use of NADPH in stoichiometric quantity, its regeneration can be promoted by combining the ER reduction with the oxidation of glucose catalysed by a glucose dehydrogenase (e.g., GDH from *Bacillus megaterium*). The reduction of the carbonyl group of ketone **3** can be performed in the presence of an ADH, using NADH or NADPH as a cofactor, and the same enzymatic system for cofactor regeneration.

### 2.2. Selection of Ene-Reductases and Alcohol Dehydrogenases with the Desired Stereoselectivity

Recently, we investigated the ER-mediated reduction of unsaturated ketone **2** within a work devoted to studying the different stereochemical courses of the hydrogenation of certain activated alkenes catalysed by Old Yellow Enzyme 1 from *Saccharomyces pastorianus* (OYE1) and Old Yellow Enzyme 3 from *S. cerevisiae* (OYE3) [23]. Although the sequence of OYE3 is 80% identical to that of OYE1, the reduction of the C=C double bond of ketone **2** afforded (*S*)-**3** (conversion = c > 98%, enantiomeric excess = ee = 78%) in the presence of OYE3, whereas (*R*)-**3** was obtained (c > 98%, ee = 44%) when OYE1 was employed as a catalyst (Table 1). The investigation was also extended to the use of the complete sets of W116 mutants of OYE1 and OYE3, because the amino acid residue in this position had been recognized to be strategic in influencing the substrate binding mode in the active site of these enzymes [24]. In particular, our screening showed that the variant OYE1-W116V was able to completely convert **2** into (*S*)-**3** showing ee = 86%.

With the aim of selecting the best biocatalysts for the first step of the synthetic procedure for this work, we also considered other ERs for the C=C double bond reduction of compound **2**. The results are reported in Table 1.

This new screening highlighted that compound **2** is well-accepted by ERs, but its reduction generally occurs with low enantioselectivity, with the exception of OYE2.6 (from *Pichia stipitis*). This enzyme could be chosen to perform the first step of the biocatalysed synthesis of the (4*R*)-stereoisomers of pheromone **1**. As for the (*S*)-selective reduction, the W116V mutant of OYE1 was selected. (*S*)-**3** is itself an interesting compound, because it is the principal alarm pheromone of the ant *Atta texana* [25].

**Scheme 2:**
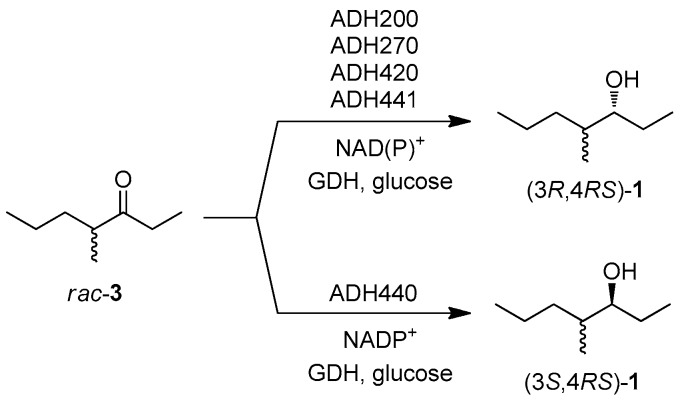
ADH screening on racemic **3** (substrate 5 mM, ADH, GDH, glucose, NAD^+^, NADP^+^ potassium phosphate buffer 50 mM pH 7.0, 30 °C, 24 h). The stereoisomeric composition of the products were obtained by GC analysis of the corresponding acetyl derivatives on a chiral stationary phase.

For the second step of the synthetic sequence, a set of 18 commercial ADHs (from EVOXX) were screened to identify the enzymes capable of reducing the carbonyl group of ketone **3**, and creating the new stereogenic centre with either (*S*) or (*R*) absolute configuration. The preliminary screening was performed on racemic **3**: five ADHs were able to promote the carbonyl reduction and the results are collected in Scheme 2. ADH440 was the only catalyst showing (*S*)-selectivity, and among the pro-(*R*)-enzymes ADH270 was selected for the optimization of the one-pot synthetic procedure.

### 2.3. One-Pot Multi-Enzymatic Synthesis of the Four Stereoisomers of 4-Methylheptan-3-ol

**Scheme 3:**
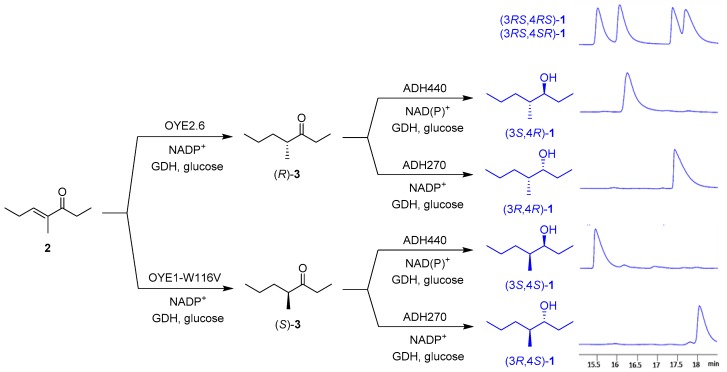
One-pot multi-enzymatic conversion of **2** into the four stereoisomers of **1**.

The one-pot sequential enzymatic synthesis of the four stereoisomers of **1** was performed on 100 mg scale in potassium phosphate buffer solution (pH 7.0) at 30 °C, using DMSO as a cosolvent for unsaturated ketone **2**. The suitable pair of enzymes (OYE2.6/ADH440, OYE2.6/ADH270, OYE1-W116V/ADH440, OYE1-W116V/ADH270) were added sequentially to the reaction medium in the presence of the same cofactor regeneration system for both ER and ADH (NADP^+^, GDH, glucose) (Scheme 3).

Extraction of the reaction mixture with EtOAc and purification of the residue by column chromatography afforded the four stereoisomers of compound **1** in good isolation yields (72–83%) and excellent stereoselectivity: (3*S*,4*R*)-**1** ee = 99%, de = 99%; (3*R*,4*R*)-**1** ee = 99%, de = 99%; (3*S*,4*S*)-**1** ee = 99%, de = 94%; (3*R*,4*S*)-**1** ee = 99%, de = 92%.

The relative configuration of the four stereoisomers was established by comparison of their ^1^H-NMR spectra with those already described in the literature (see Materials and Methods). Once the relative configuration was defined, the absolute configuration was assigned by comparing the optical rotation values of the samples with those reported in the literature (see Materials and Methods).

## 3. Discussion

This synthetic procedure shows remarkable improvements with respect to those already reported in the literature, especially in the light of the so-called “twelve principles” of green chemistry [26]. Most of the reagents are incorporated into the final product, and a reduced quantity of non-hazardous waste is generated: water is produced as a by-product of the aldolic condensation affording compound **2**, and the two enzymatic reductions occur at the expense of the oxidation of glucose to gluconic acid.

Biocatalysts from renewable feedstocks are employed instead of stoichiometric reagents. The combination of suitable proteins enables the complete control of the absolute configuration of the two stereocentres, in order to produce all the possible stereoisomers without the use of complex derivatization strategies. The high selectivity of the enzymatic reductions makes the one-pot procedure possible, thus avoiding the work-up of intermediate products and reducing the waste of organic solvents. The final result is a three-step sequence, starting from diethylketone and propanal, to afford high-value chiral products with the creation of two vicinal stereogenic centres under enzymatic control.

## 4. Materials and Methods

GC-MS analyses were performed using a HP-5MS column (30 m × 0.25 mm × 0.25 µm, Agilent, Santa Clara, CA, USA). The following temperature program was employed: 60 °C (1 min)/6 °C·min^−1^/150 °C (1 min)/12 °C·min^−1^/280 °C (5 min). ^1^H and ^13^C NMR spectra were recorded on a 400 or 500 MHz spectrometer (Bruker, Billerica, MA, USA) and the chemical shift scale was based on internal tetramethylsilane. All the chromatographic separations were carried out on silica gel. Chiral GC analyses of compounds **1** (as acetyl derivatives obtained by treatment with acetic anhydride in pyridine) and **3** were performed on a Chirasil DEX CB (25 m × 0.25 mm × 0.25 μm, Chrompack, Agilent, Santa Clara, CA, USA) column, installed on HP 6890 gas chromatograph (Agilent, Santa Clara, CA, USA); (a) compound **1** 45 °C/1.0 °C·min^−1^/65 °C (1 min)/50 °C·min^−1^/180 °C (5 min): (3*S*,4*S*)-**1** t_R_ = 15.5 min, (3*S*,4*R*)-**1** t_R_ = 16.0 min, (3*R*,4*R*)-**1** t_R_ = 17.3 min, (3*R*,4*S*)-**1** t_R_ = 17.7 min; (b) compound **3**: 55 °C/0.8 °C·min^−1^/67 °C (1 min)/90 °C min^−1^/180 °C·2 min), (*R*)-**3** t_R_ = 5.0 min, (*S*)-**3** t_R_ = 5.5 min.

### 4.1. Strains and Enzymes

A screening kit of 18 ADHs was purchased from EVOXX (Monheim am Rhein, Germany). Ene-reductases (OYE1-3, OYE1-W116V, OYE2.6, YqjM, and LeOPR1) and glucose dehydrogenase (GDH) were overproduced in *E. coli* BL21(DE3) strains harbouring a specific plasmid prepared as previously reported: pET30a-OYE1 from the original plasmid provided by Neil C. Bruce [27], pET30a-OYE2 and pET30a-OYE3 from *S. cerevisiae* BY4741 and pKTS-GDH from *B. megaterium* DSM509 [28]; pDJBx-OYE2.6, pDJB5-OYE1-W116V, and pDJBx-LeOPR1 from the original plasmids provided by Prof. Jon D. Stewart. For YqjM, the original plasmid provided by Prof. M. Hall was used directly as provided [29]. The enzymes were produced and purified as described in Section 4.2.

### 4.2. Overproduction of Enzymes in E. coli BL21(DE3)

LB medium (5 mL) containing the appropriate antibiotic (50 μg·mL^−1^ kanamycin for pET30a, 100 μg·mL^−1^ ampicillin for pKTS, pDJBx, 30 µg mL^−1^ chloramphenicol for YqjM) was inoculated with a single colony from a fresh plate and grown for 8 h at 37 °C and 220 rpm. This starter culture was used to inoculate 200 mL LB medium (TB medium in the case of YqjM), which was incubated for 8 h under the same conditions and used to inoculate 1.5 L medium. The latter culture was shaken at 37 °C and 220 rpm until OD_600_ reached 0.4–0.5, then enzyme expression was induced by the addition of 0.1 mM IPTG (50 ng·mL^−1^ anhydrotetracycline was also added in the case of the pKTS-GDH plasmid).

In the case of OYE1-3, GDH, and YqjM, after 5–6 h the cells were harvested by centrifugation (5000× *g*, 20 min, 4 °C), resuspended in 50 mL of lysis buffer (20 mM KP_i_ buffer pH 7.0, 300 mM NaCl, 10 mM imidazole) and disrupted by sonication (Omni Ruptor 250 ultrasonic homogeniser, Omni International Inc., Kennesaw, GE, USA, five sonication cycles, 15 s each, 50% duty). YqjM was used as cell-free extract, whereas for OYE1-3 and GDH the cell-free extract after centrifugation (20,000× *g*, 20 min, 4 °C) was chromatographed on IMAC stationary phase (Ni-Sepharose Fast Flow, GE Healthcare, Little Chalfont, UK) with a mobile phase composed of 20 mM KP_i_ buffer pH 7.0, 300 mM NaCl, and a 10–300 mM imidazole gradient. Protein elution was monitored at 280 nm, and the fractions were collected according to the chromatogram and dialysed twice against 1.0 L of 50 mM KP_i_ buffer pH 7.0 (12 h each, 4 °C) to remove imidazole and salts. Purified protein aliquots were stored frozen at −80 °C.

In the case of OYE2.6 and LeOPR1—produced as Glutathione *S*-transferase (GST)-fusion proteins—cell pellets were resuspended in cold sepharose binding buffer (PBS, 140 mM NaCl, 2.7 mM KCl, 10 mM Na_2_HPO_4_, 1.8 mM KH_2_PO_4_, pH 7.3) and lysed by sonication (Omni Ruptor 250 ultrasonic homogeniser, five sonication cycles, 15 s each, 50% duty). The cell-free extract was centrifuged (12,000 rpm, 40 min, 4 °C). The resulting supernatant was passed through Glutathione Sepharose 4 Fast Flow (GE Healthcare), with PBS buffer as the mobile phase. Once the absorbance (280 nm) returned to a baseline reading, the desired protein was eluted by adding a reduced glutathione (GSH) buffer solution (10 mM γ-l-glutamyl-l-cysteinylglycine, 50 mM Tris-HCl, pH 8.0). Protein elution was monitored at 280 nm and the fractions were collected according to the chromatogram. Purified protein aliquots were stored frozen at −80 °C.

### 4.3. One-Pot Conversion of ***2*** into the Four Stereoisomers of ***1***

A solution of unsaturated ketone **2** (100 mg, 0.794 mmol) in DMSO (500 μL) was added to a potassium phosphate buffer solution (100 mL, 50 mM, pH 7.0) containing the suitable OYE (10–15 mg) (two batches with OYE2.6 and two batches with OYE1-W116V), glucose (4 eq., 3.18 mmol, 572 mg), GDH (125 U), and NADP^+^ (0.025 eq., 20 μmol, 15 mg). The reactions were incubated for 24 h in an orbital shaker at 30 °C. The intermediates (*R*) and (*S*)-**3** (ee = 99% and 92%, respectively, in both batches) were not isolated, and were submitted to carbonyl reduction. ADH440 or ADH270 (5 mg, two batches each), glucose (1 eq., 0.794 mmol, 143 mg), and GDH (125 U) were added to the four reaction mixtures. After 24 h incubation at 30 °C, the final products were recovered and purified by column chromatography, eluting with hexane and increasing quantities of EtOAc.

#### 4.3.1. (3*R*,4*R*)-4-Methylheptan-3-ol ((3*R*,4*R*)-**1**)

From **2** (100 mg, 0.794 mmol), after reduction with OYE2.6 and ADH270, compound (3*R*,4*R*)-**1** (85.7 mg, 83%) was obtained: ee = 99% (chiral GC), de = 99% (^1^H-NMR); [α]_D_ = + 23.0 (*c* 1.2, hexane) [lit. [9] [α]_D_ = +22.7 (*c* 0.264, hexane, for (3*R*,4*R*)-**1** ee = 98%, de = 99%)]; ^1^H-NMR (CDCl_3_, 400 MHz) [19] *δ* 3.41 (dt, *J* = 8.4 and 4.3 Hz, *CH*OH), 1.60–1.00 (8H, m, 3*CH_2_*+*CH+OH*), 0.95 (3H, t, *J* = 7.4 Hz, *CH_3_*CH_2_), 0.90 (3H, t, *J* = 7.1 Hz, *CH_3_*CH_2_), 0.86 (3H, d, *J* = 6.8 Hz, *CH_3_*CH); ^13^C-NMR (CDCl_3_, 125 MHz) [19] *δ* 76.9, 37.6, 35.8, 27.4, 20.6, 14.5, 13.6, 10.7; GC/MS (EI) *m*/*z*: t_R_ S= 4.98 min, 112 (M^+^ − 18, 1), 101 (16), 83 (25), 59 (100).

#### 4.3.2. (3*S*,4*R*)-4-Methylheptan-3-ol ((3*S*,4*R*)-**1**)

From **2** (100 mg, 0.794 mmol), after reduction with OYE2.6 and ADH440, compound (3*S*,4*R*)-**1** (78.4 mg, 76%) was obtained: ee = 99% (chiral GC), de = 99% (^1^H-NMR); [α]_D_ = +11.5 (*c* 1.0, hexane) [lit. [16] [α]_D_ = +10.9 (*c* 1.87, hexane, for (3*S*,4*R*)-**1** ee = 92%, de = 99%)]; ^1^H-NMR (CDCl_3_, 400 MHz) [19] *δ* 3.35 (ddd, *J* = 8.7, 5.2, and 3.5 Hz, *CH*OH), 1.60–1.00 (8H, m, 3*CH_2_*+*CH+OH*), 0.96 (3H, t, *J* = 7.4 Hz, *CH_3_*CH_2_), 0.90 (3H, t, *J* = 7.1 Hz, *CH_3_*CH_2_), 0.88 (3H, d, *J* = 6.8 Hz, *CH_3_*CH); ^13^C-NMR (CDCl_3_, 125 MHz) [19] *δ* 77.8, 38.4, 34.3, 26.4, 20.5, 15.5, 14.5, 10.5; GC/MS (EI) *m*/*z*: t_R_ = 5.02 min, 113 (M^+^ − 17, 1), 101 (16), 83 (20), 59 (100).

#### 4.3.3. (3*R*,4*S*)-4-Methylheptan-3-ol ((3*R*,4*S*)-**1**)

From **2** (100 mg, 0.794 mmol), after reduction with OYE1-W116V and ADH270, compound (3*R*,4*S*)-**1** (83.6 mg, 81%) was obtained: ee = 99%(chiral GC), de = 92% with respect to (3*R*,4*R*)-**1** (^1^H-NMR); [α]_D_ = −10.7 (*c* 1.4, hexane) [lit. [16] [α]_D_ = +10.9 (*c* 1.87, hexane, for (3*S*,4*R*)-**1** ee = 92%, de = 99%)]; spectroscopic data were in agreement with those of the enantiomer.

#### 4.3.4. (3*S*,4*S*)-4-Methylheptan-3-ol ((3*S*,4*S*)-**1**)

From **2** (100 mg, 0.794 mmol), after reduction with OYE1-W116V and ADH440, compound (3*S*,4*S*)-**1** (74.3 g, 72%) was obtained: ee = 99% (chiral GC), de = 94% with respect to (3*S*,4*R*)-**1** (^1^H-NMR); [α]_D_ = −18.9 (*c* 1.1, hexane) [lit. [9] [α]_D_ = +22.7 (*c* 0.264, hexane, for (3*R*,4*R*)-**1** ee = 98%, de = 99%)]; the spectroscopic data were in agreement with those of the enantiomer.

## Figures and Tables

**Table 1:**
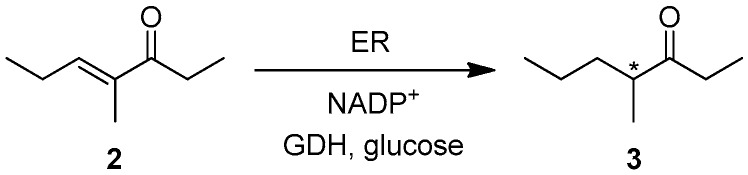
Ene-reductase-mediated hydrogenation ^1^ of unsaturated ketone **2**.

ER	c (%) ^2^	ee (%) ^3^
OYE1 ^4^	99	44, *R*
OYE3 ^4^	99	78, *S*
OYE1-W116V ^4^	99	86, *S*
OYE2	99	40 *R*
OYE2.6	99	99, *R*
LeOPR1	99	70, *R*
YqjM	99	60, *S*
PETN	99	22, *R*

^1^ Substrate (5 mM), glucose (20 mM), Old Yellow Enzyme (OYE), glucose dehydrogenase (GDH), NADP^+^, DMSO (1%), phosphate buffer pH 7.0, 24 h; see Table S1 in the Supplementary Information for data concerning the ERs employed in this screening; ^2^ Conversion calculated on the basis of GC analysis of the crude mixture after 24 h; ^3^ Enantiomeric excess calculated on the basis of GC analysis (see Materials and Methods) on a chiral stationary phase; ^4^ ref. [23].

## Supplementary Materials

The following are available online: synthesis and characterization of compound **2**, synthesis and characterization of compounds (*S*) and (*R*)-**3**, general procedure for ER-mediated reduction of unsaturated ketone **2** (screening), general procedure for ADH-mediated reduction of racemic **3** (screening), representative GC chromatograms on chiral stationary phases, Table S1.
