# Supplementary material for: One-Pot Multi-Enzymatic Synthesis of the Four Stereoisomers of 4-Methylheptan-3-ol
Source: Molecules. 2017 Sep 22;22(10):1591. doi: 10.3390/molecules22101591 (PMC6151462; doi:10.3390/molecules22101591)
Supplement: Supplementary file 1 [file molecules-22-01591-s001.pdf]

Supplementary information

One-pot multi-enzymatic synthesis of the four stereoisomers of 4-methylheptan-3-ol

Elisabetta Brenna,*[1] Michele Crotti,[1§] Francesco G. Gatti,[1] Daniela Monti,[2] Fabio Parmeggiani,[1] Andrea Pugliese[1]

[1] Politecnico di Milano, Dipartimento di Chimica, Materiali, Ingegneria Chimica, Via Mancinelli 7, I-20131 Milano, Italy.
[2] Istituto di Chimica del Riconoscimento Molecolare – CNR, Via M. Bianco 9, I-20131 Milano, Italy
* Correspondence: mariaelisabetta.brenna@polimi.it; Tel.: +39 0223993077
§ Authors are listed in alphabetical order. M. C. is the principal author of the publication.

Contents **Page**

General methods S2
(*E*)-4-Methylhept-4-en-3-one (**2**) S2
(*S*)-4-Methylheptan-3-one ((*S*)-**3**) S3
(*R*)-4-Methylheptan-3-one ((*R*)-**3**) S3
General procedure for ER-mediated reduction of unsaturated ketone **2** (screening) S4
General procedure for ADH-mediated reduction of racemic **3** (screening) S4
Representative GC chromatograms on chiral stationary phases S5
Table S1 .... S7
References. S7

General methods

GC-MS analyses were performed using a HP-5MS column (30 m × 0.25 mm × 0.25 μm, Agilent). The following temperature program was employed: 60°C (1 min) / 6°C min^{-1} / 150°C (1 min) / 12°C min^{-1} / 280°C (5 min). ^{1}H and ^{13}C NMR spectra were recorded on a 400 or 500 MHz spectrometer and the chemical shift scale was based on internal tetramethylsilane. All the chromatographic separations were carried out on silica gel columns. Chiral GC analyses of compound **3** were performed on a Chirasil DEX CB (25 m × 0.25 mm × 0.25 μm, Chrompack) column, installed on HP 6890 gas chromatograph: 55°C / 0.8°C min^{-1} / 67°C (1 min) / 90°C min^{-1} / 180°C (2 min), (*R*)-**3** t_R = 5.0 min, (*S*)-**3** t_R = 5.5 min.

(*E*)-4-Methylhept-4-en-3-one (2)

A solution of KOH in MeOH (2 mL, 3 M) was added to diethylketone (7.74 g, 0.090 mol). The mixture was cooled to 4°C and propanal (2.61 g, 0.045 mol) was added dropwise. The reaction was stirred at room temperature overnight. Unreacted diethylketone was removed by distillation under reduced pressure and the residue was poured into diluted HCl and extracted with EtOAc. The organic phase was dried over anhydrous Na_2SO_4 and concentrated *in vacuo*, to give a residue (ca. 3.5 g) which was submitted to dehydration by treatment with oxalic acid (0.40 g) in refluxing toluene (50 mL). The mixture was poured into water and extracted with EtOAc. The organic phase was dried over anhydrous Na_2SO_4 and concentrated *in vacuo*, to give a residue which was purified by column chromatography, eluting with *n*-hexane and increasing quantity of EtOAc, to afford unsaturated ketone **2** (3.18 g, 56%): ^{1}H-NMR ($CDCl_3$, 400 MHz) [S1]: δ (ppm) 6.61 (1H, t, J = 7.2 Hz, *CH*=), 2.68 (2H, q, J = 7.3 Hz, CO$CH_2$$CH_3$), 2.25 (2H, quintuplet, J = 7.3 Hz, CH_2CH=), 1.78 (3H, s, CH_3C=), 1.13 – 1.03 (6H, m, $CH_3$$CH_2$CO and $CH_3$$CH_2$C=); ^{13}C-NMR ($CDCl_3$, 100.6 MHz): δ (ppm) 202.6, 143.4, 136.6, 30.4, 22.4, 13.2, 11.3, 8.9; GC-MS (EI) m/z (%) = 126 (M^+, 20), 97 (100), 69 (90).

(*S*)-4-Methylheptan-3-one ((*S*)-3)

A solution unsaturated ketone **2** in DMSO (1 mL, 400 mM, 0.4 mmol) was added to a potassium phosphate buffer solution (25 mL, 50 mM, pH 7.0) containing OYE1-W116V (5 mg), GDH (100 U), glucose (4 eq, 1.6 mmol, 288 mg) and $NADP^+$ (0.02 eq, 8 µmol, 5.9 mg). The reaction was incubated for 24 h in an orbital shaker at 30°C. The mixture was then extracted with EtOAc (3 × 10 mL), dried over anhydrous Na_2SO_4 and purified by column chromatography (*n*-hexane with increasing amount of EtOAc) to afford compound (*S*)-**3**: ee = 92% (chiral GC); $[\alpha]_D$ = + 20.8 (*c* 1.5, hexane) [lit. [S2] $[\alpha]_D$ = + 21.0 (*c* 1, hexane) for (*S*)-**3]; ^{1}H-NMR ($CDCl_3$, 400 MHz) [S3]: δ (ppm) 2.58 – 2.50 (1H, m, CO*CH*), 2.45 (2H, qd, *J* = 7.4 and 2.5 Hz, CH_2CH_3); 1.68 – 1.55 (1H, m, CH*CH*HCH$_2$), 1.38 – 1.20 (3H, m, CH*CH*HCH$_2$ + CH_2CH_3), 1.06 (3H, d, *J* = 7.0 Hz, CH_3CH), 1.04 (3H, t, *J* = 7.1 Hz, CH_3CH_2CO), 0.90 (3H, t, *J* = 7.6 Hz, $CH_3CH_2CH_2$); ^{13}C-NMR ($CDCl_3$, 100.6 MHz) [S3]: δ (ppm) 215.5, 46.0, 35.4, 34.3, 20.6, 16.5, 14.2, 7.9; GC-MS (EI) m/z (%) = 128 (5), 86 (49), 71 (74), 57 (100).

(*R*)-4-Methylheptan-3-one ((*R*)-3)

A solution unsaturated ketone **2** in DMSO (1 mL, 400 mM, 0.4 mmol) was added to a potassium phosphate buffer solution (25 mL, 50 mM, pH 7.0) containing OYE2.6 (5 mg), GDH (100 U), glucose (4 eq, 1.6 mmol, 288 mg) and $NADP^+$ (0.02 eq, 8 µmol, 5.9 mg). The reaction was incubated for 24 h in an orbital shaker at 30°C. The mixture was then extracted with EtOAc (3 × 10 mL), dried over anhydrous Na_2SO_4 and purified by column chromatography (*n*-hexane with increasing amount of EtOAc) to afford compound (*S*)-**3**: ee = 99% (chiral GC); $[\alpha]_D$ = - 21.9 (*c* 1.2, hexane). The spectroscopic data were in agreement with those of the enantiomer.

General procedure for ER-mediated reduction of unsaturated ketone 2 (screening)

A solution of unsaturated ketone **2** in DMSO (10 μL, 500 mM, 5 μmol) was added to a potassium phosphate buffer solution (1.0 mL, 50 mM, pH 7.0) containing glucose (20 μmol), $NADP^+$ (0.1 μmol), GDH (4 U) and the required purified or cell-free extract OYE (80-120 μg). The mixture was incubated for 24 h in an orbital shaker (160 rpm, 30°C). The solution was extracted with EtOAc (2 × 250 μL), centrifuging after each extraction (15000 *g*, 1.5 min), and the combined organic solutions were dried over anhydrous Na_2SO_4. Two replicates were performed for each biotransformation: no significant differences (less than 5%) were observed for conversion and enantiomeric excess values.

General procedure for ADH-mediated reduction of racemic 3 (screening)

A solution of ketone **3** in DMSO (10 μL, 500 mM, 5 μmol) was added to a potassium phosphate buffer solution (1.0 mL, 50 mM, pH 7.0) containing glucose (20 μmol), $NADP^+$ (0.1 μmol) or NAD^+ (0.1 μmol) (according to the ADH preference), GDH (4 U) and the required ADH (200 μg). The mixture was incubated for 24 h in an orbital shaker (160 rpm, 30°C). The solution was extracted with EtOAc (2 × 250 μL), centrifuging after each extraction (15000 g, 1.5 min), and the combined organic solutions were dried over anhydrous Na_2SO_4. The sterereoisomeric composition of the products was determined by GC analysis of the corresponding acetyl derivatives on a chiral column. Two replicates were performed for each biotransformation: no significant differences (less than 5%) were observed for conversion and stereoselectivity values.

Representative GC chromatograms on chiral stationary phases

4-Methylheptan-3-one (**3**)

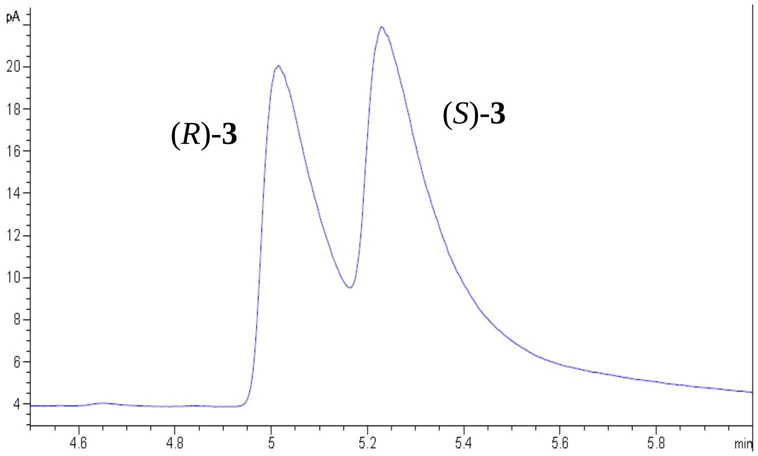

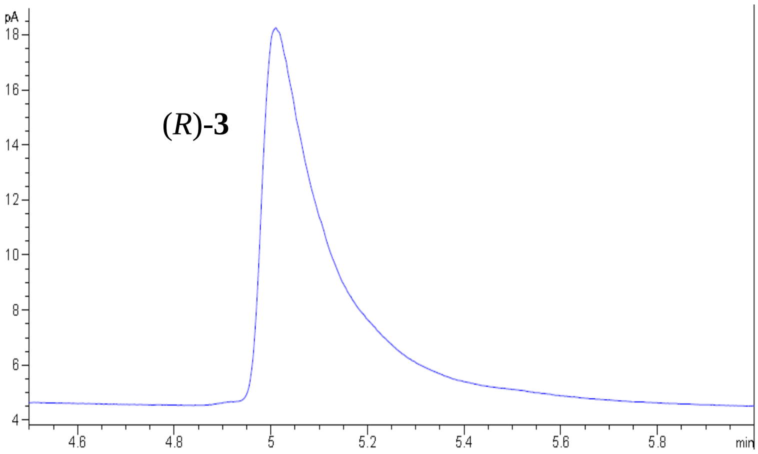

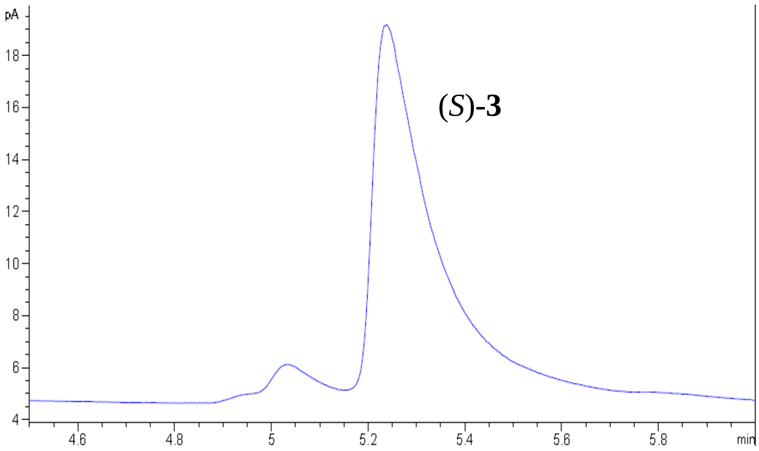

4-Methylheptan-3-ol (**1**)

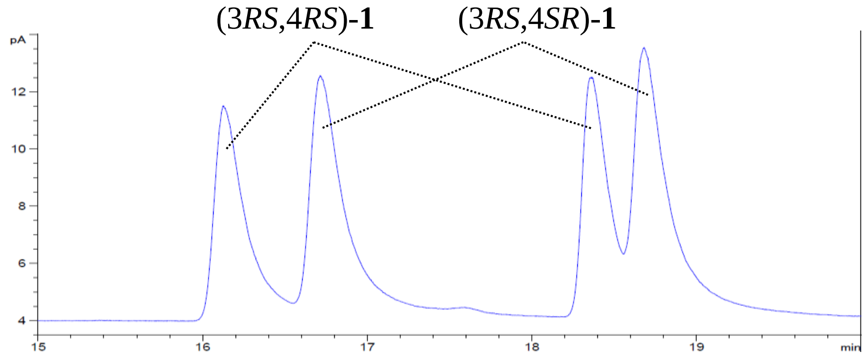

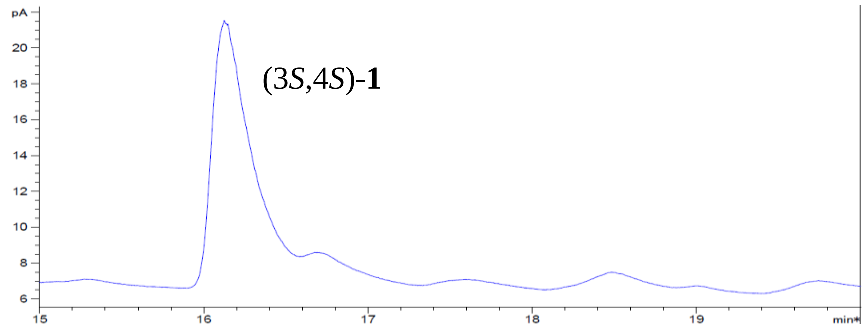

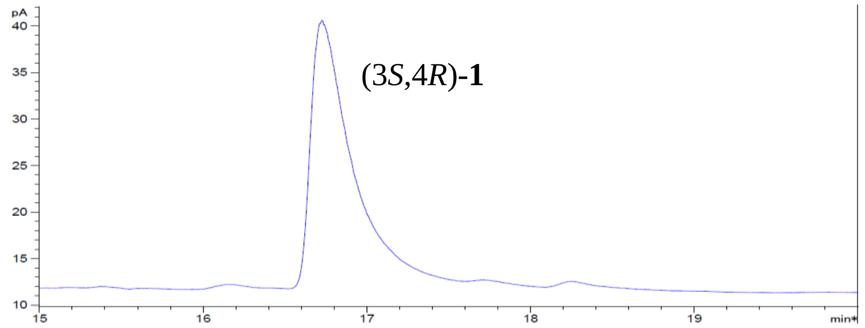

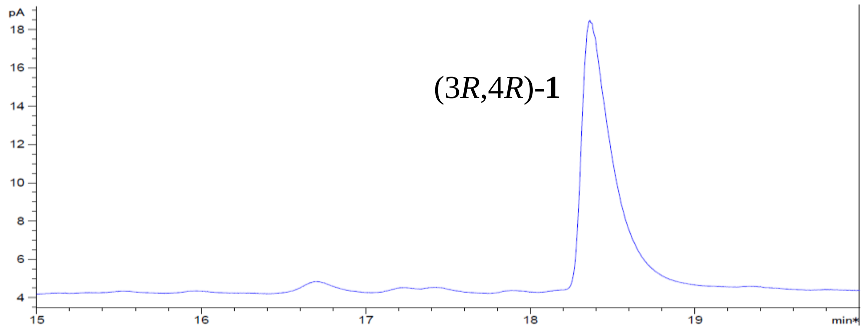

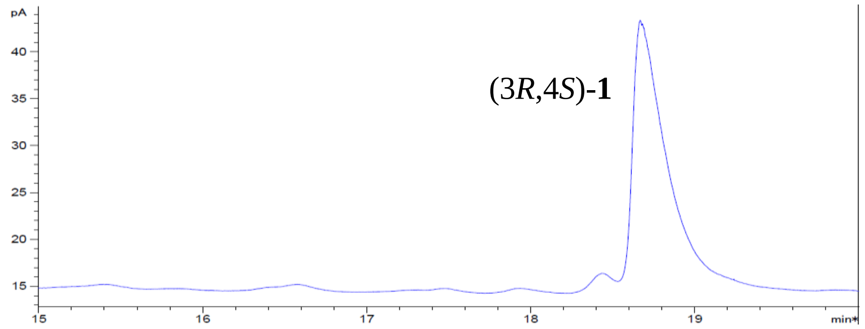

Table S1. Source and literature data for the panel of ERs employed in this work.

ER	Organism	Annotated function	UniProt	Ref.
OYE1	*Saccharomyces pastorianus* (formerly *S. carlsbergensis*)	NAD(P)H dehydrogenase	Q02899	[S4]
OYE2	*Saccharomyces cerevisiae*	NAD(P)H dehydrogenase	Q03558	[S5]
OYE3	*Saccharomyces cerevisiae*	NAD(P)H dehydrogenase	P41816	[S6]
OYE2.6	*Scheffersomyces stipitis* (formerly *Pichia stipitis*)	NAD(P)H dehydrogenase	A3LT82	[S7]
LeOPR1	*Solanum lycopersicum* (formerly *Lycopersicon esculentum*)	12-oxophytodienoate reductase	Q9XG54	[S8]
YqjM	*Bacillus subtilis*	NAD(P)H dehydrogenase	P54550	[S9]
PETNR	*Enterobacter cloacae*	pentaerythritol tetranitrate reductase	P71278	[S10]

References

[S1] Liu, J.; Ma, S. *Org. Lett.* **2013**, *15*, 5150-5153.

[S2] Riley, R. G.; Silverstein, R. M. *Tetrahedron* **1974**, *30*, 1171-1174.

[S3] Wang, L.; Xu, Z., Ye, T. *Org. Lett.* **2011**, *13*, 2506-2509.

[S4] Saito, K.; Thiele, D. J.; Davio, M.; Lockridge, O.; Massey, V. *J. Biol. Chem.* **1991**, *266*, 20720-20724.

[S5] Stott, K.; Saito, K.; Thiele, D. J.; Massey, V. *J. Biol. Chem.* **1993**, *268*, 6097-6106.

[S6] Niino, Y. S.; Chakraborty, S.; Brown, B. J.; Massey, V. *J. Biol. Chem.* **1995**, *270*, 1983-1991.

[S7] Pompeu, Y. A.; Sullivan, B.; Walton, A. Z.; Stewart, J. D. *Adv. Synth. Catal.* **2012**, *354*, 1949-1960.

[S8] Fitzpatrick, T. B.; Amrhein, N.; Macheroux, P. *J. Biol. Chem.* **2003**, *278*, 19891-19897.

[S9] Strassner, J.; Fuerholz, A.; Macheroux, P.; Amrhein, N.; Schaller, A. *J. Biol. Chem.* **1999**, *274*, 35067-35073.

[S10] French, C. E.; Nicklin, S.; Bruce, N. C. *J. Bacteriol.* **1996**, *178*, 6623-6627.
